# Real-time 4K computer-generated hologram based on encoding conventional neural network with learned layered phase

**DOI:** 10.1038/s41598-023-46575-1

**Published:** 2023-11-08

**Authors:** Chongli Zhong, Xinzhu Sang, Binbin Yan, Hui Li, Xinhui Xie, Xiujuan Qin, Shuo Chen

**Affiliations:** 1 State Key Laboratory of Information Photonics and Optical Communications, Beijing University of Posts and Telecommunications, Beijing, 100876 China; 2 Beijing National Research Center for Information Science and Technology, Tsinghua University, Beijing, 100084 China; 3 Beijing Institute of Control and Electronic Technology, Beijing, 100038 China

**Keywords:** Displays, Imaging and sensing

## Abstract

Learning-based computer-generated hologram (CGH) demonstrates great potential for real-time high-quality holographic displays. However, real-time 4K CGH generation for 3D scenes remains a challenge due to the computational burden. Here, a variant conventional neural network (CNN) is presented for CGH encoding with learned layered initial phases for layered CGH generation. Specifically, the CNN predicts the CGH based on the input complex amplitude on the CGH plane, and the learned initial phases act as a universal phase for any target images at the target depth layer. These phases are generated during the training process of the coding CNN to further optimize the quality. The CNN is trained to learn encoding 3D CGH by randomly selecting the depth layer in the training process, and contains only 938 parameters. The generation time for a 2D 4K CGH is 18 ms, and is increased by 12 ms for each layer in a layered 3D scene. The average Peak Signal to Noise Ratio (PSNR) of each layer is above 30dB in the depth range from 160 to 210 mm. Experiments verify that our method can achieve real-time layered 4K CGH generation.

## Introduction

Computer-generated hologram (CGH) has been actively researched in recent years, owing to its ability to provide ideal depth cues for 3D display applications^1,2^. Traditional CGH generation simulates the diffraction from the 3D objects to the CGH plane with a computer, and the complex amplitude is then encoded into the CGH. The encoded CGH can be uploaded onto a spatial light modulator (SLM), which modulates the incident light into the target complex amplitude^3,4^. Finally, the 3D scene can be reconstructed through diffraction.

Many approaches have been proposed to accelerate the CGH generation and improve its quality^5–9^. High-quality CGH generation is a time-consuming process, and advances in this area have been achieved by integrating deep learning techniques^10–12^. Conventional neural networks (CNN), such as U-Net, are ideal for CGH prediction due to their high-resolution and high-speed capabilities^13^. Nonetheless, real-time 4K holographic displays remains a significant challenge for learning-based CGH algorithms due to the high computational burden.

Learning-based CGH algorithms have been used to reconstruct point-based and layer-based scenes in recent research. Neural networks can play different roles in the CGH generation process, either predicting the CGH directly or serving as a module within the traditional CGH generation process.

For point-based reconstruction, precalculated CGH supervision is required in current researches. A RGBD representation is used in Tensor Holography for point-based reconstruction^14^. A CNN predicts the complex amplitude in the mid-point wavefront-recording-plane (WRP), which is supervised by the precalculated ones^15^. After diffraction calculation from the WRP to the CGH plane, the double-phase method is used for encoding CGH. The point-based reconstruction provides high depth resolution, but pre-calculation of a dataset is required. Enlarging the depth range of reconstruction also requires a larger receptive field to match the physical propagation, and the computation burden is increased if more convolution layers are used.

For layer-based reconstruction, self-supervision is supported with the input image, but CGH generation for multiple layers with high efficiency is challenging. HoloNet can generate 2D CGH at high-speed in Neural Holography^11^. A CNN predicts the phase on the image plane, and another is used to encode the CGH. The reconstruction of predicted CGH is self-supervised with the input image. Neural 3D holography and Time-multiplexed Neural Holography use CNN to learn accurate diffraction to generate high-quality CGH through iterations^16,17^. However, millions of parameters used in the Neural Holography series require much GPU resources, limiting the ability to generate high-speed 4K CGH. Holo-encoder is proposed to generate 4K CGH efficiently^13^. Given a target image, a single CNN predicts the CGH directly. 4K-DMD-Net is proposed to further enhance the quality using sub-pixel convolution method but the speed is reduced^18^. There are also methods using only one CNN to encode the CGH^19^, but real-time generation is not achieved. FFT modules are used to increase the quality, but the speed is much slower^20^. Instead of sacrificing speed for quality, a complex-valued CNN is used to generate CGH using tens of thousands of parameters^21^, and 4K CGH is generated in real-time. These methods can generate 2D CGH in high speed, but are not optimized for 3D scenes. Layered CGH can be generated by running multiple models, and it is not an efficient way.

In order to optimize the layered 3D reconstruction, Self-holo is proposed to use a CNN to predict the WRP with RGBD input similar to Tensor Holography, and another CNN to encode the CGH^22^. The 3D scene is divided into 3 layers for layered self-supervision. The depth range of reconstruction is also related to receptive field of the CNN for WRP prediction. In summary, using a CNN to process the input image for WRP prediction may result in constraints on the depth of field and receptive field, and generating multiple 2D CGHs can be computationally burdensome. To improve the quality, previous research has also explored various strategies involving training strategy, dataset, and loss function^23–25^. However, these approaches primarily focus on optimizing the training process and related aspects, rather than optimizing the neural network architecture. In contrast, our method takes a novel approach by optimizing the neural network architecture. We achieve this optimization by introducing a learned initial phase instead of relying on a CNN-predicted initial phase^11^. Our method can be combined with existing strategies to further enhance performance.

Here, we proposed a layered CGH generation method based on encoding CNN with learned layered phase. To generate layered 3D CGH with high efficiency, we seek to avoid using CNN to process the input image. For each depth layer, a corresponding learnable initial phase is used as a universal phase for any input images. After diffraction from the image planes to the CGH plane, the CNN predicts the CGH by taking the complex amplitude in the CGH plane as input. To generate multiple-layer CGH, the input image’s depth layer is randomly selected during the training process. Our model is verified by generating CGH for 2D reconstruction at different depth layers and generating 3D layered CGH by encoding the complex amplitude synthesized by multiple layers. The encoding CNN takes only 6.47 ms to encode a 4K CGH, and its computational burden does not increase for multiple layers because it only processes the synthesized complex amplitude. The diffraction takes 12 ms for each depth layer. Numerical reconstruction and optical experiments demonstrate that our method can achieve real-time 4K CGH generation for layered scenes.

## Results

**Figure 1:**
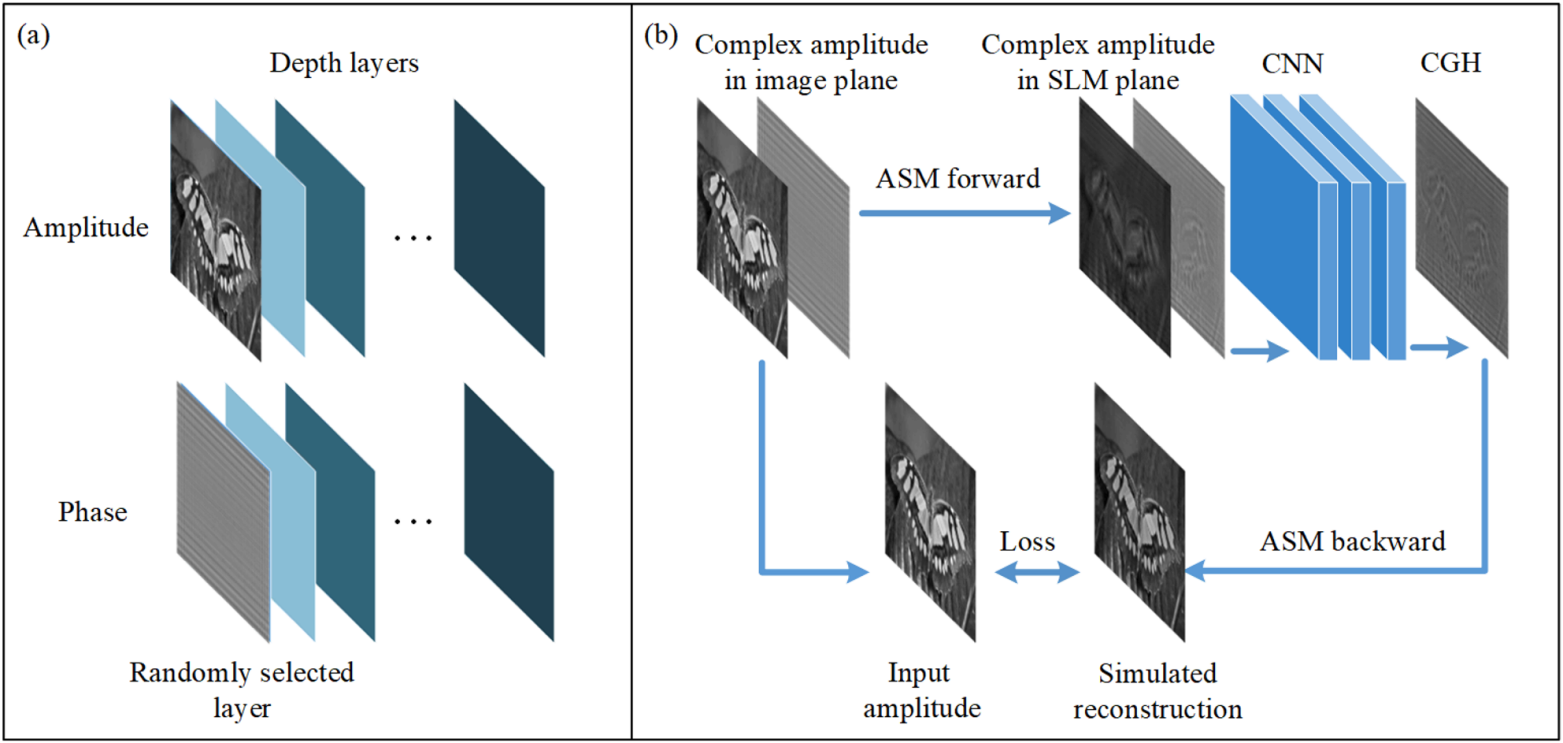
Structure of the proposed method.

### Structure and experiment results of proposed method

The structure of our method is illustrated in Fig. 1. Our model is designed to generate CGH for 2D reconstructions at different depths and layered 3D scenes. Here, 6 target depth layers are set ranging from 160 to 210 mm with an interval of 10 mm. For each depth layer, an initial zero phase is used as learnable parameters during the training process. As shown in Fig. 1a, for each input amplitude, its depth layer is selected randomly from the 6 target depth layers. The input amplitude is then given the corresponding initial phase. After diffraction from the image plane to the CGH plane with the Angular Spectrum Method (ASM), a CNN is utilized to encode the CGH. The amplitude of numerical reconstruction is then compared with the input amplitude for self-supervision. Further details of the training process are introduced in Methods section.

**Figure 2:**
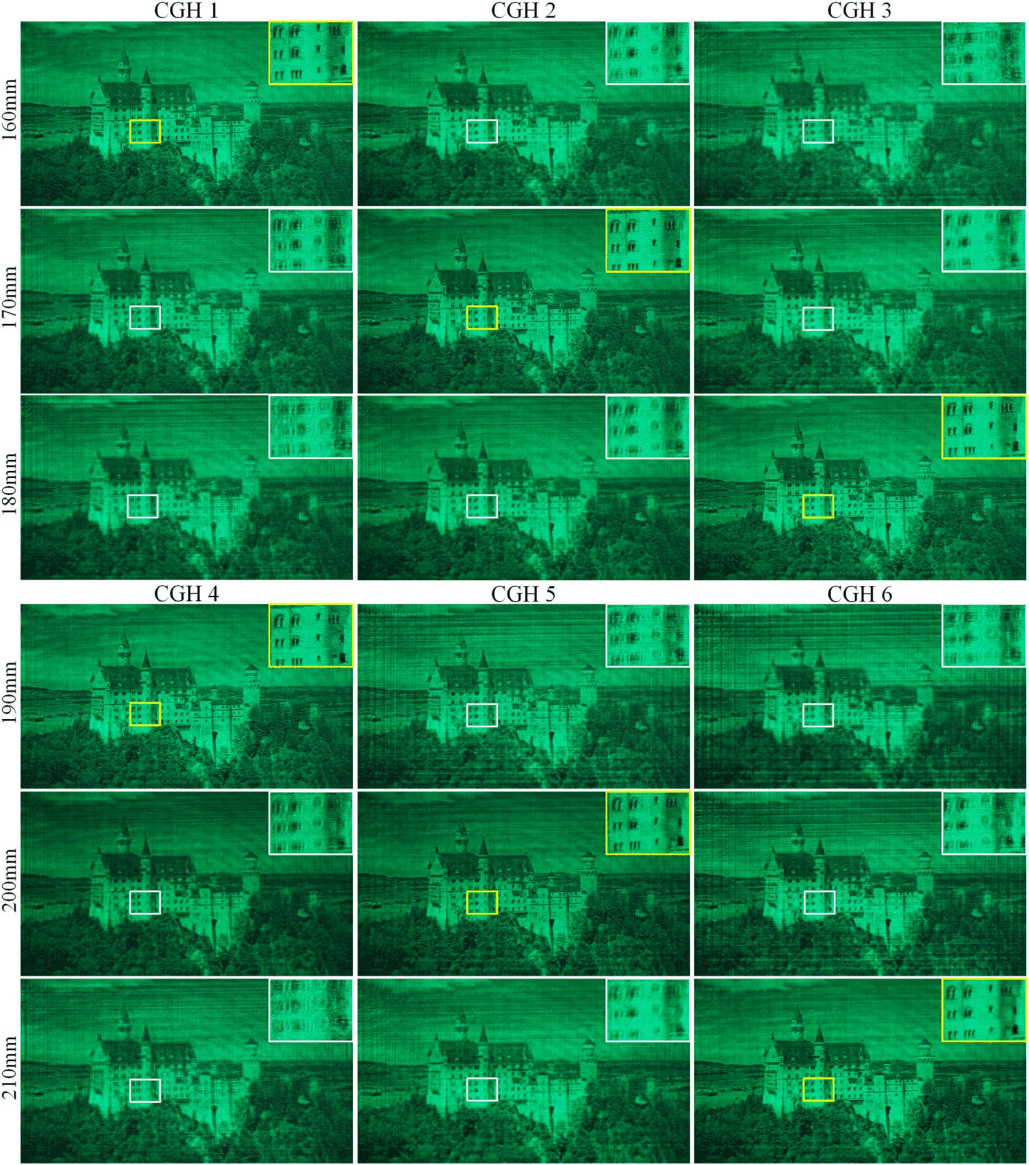
Optical reconstruction of the 6 depth layers.

Here, we present the optical reconstructions of 6 depth layers in Fig. 2. The input image is from the DIV2K validation dataset, which is not used to train our model. 6 CGHs are generated with our proposed method, corresponding to depth layers of 160 mm, 170 mm, 180 mm, 190 mm, 210 mm and 210 mm, respectively. The focused reconstruction is highlighted in yellow. The optical reconstructions are clear when the corresponding depth layer is in focus, while other reconstructions are defocused. For instance, CGH 1, 2, and 3 are generated at 160 mm, 170 mm, and 180 mm, respectively. At a depth layer of 160 mm, only the reconstruction of CGH 1 is clear, while the other reconstructions are defocused. We can see that our method can successfully generate CGHs for 2D reconstructions at different depths.

**Figure 3:**
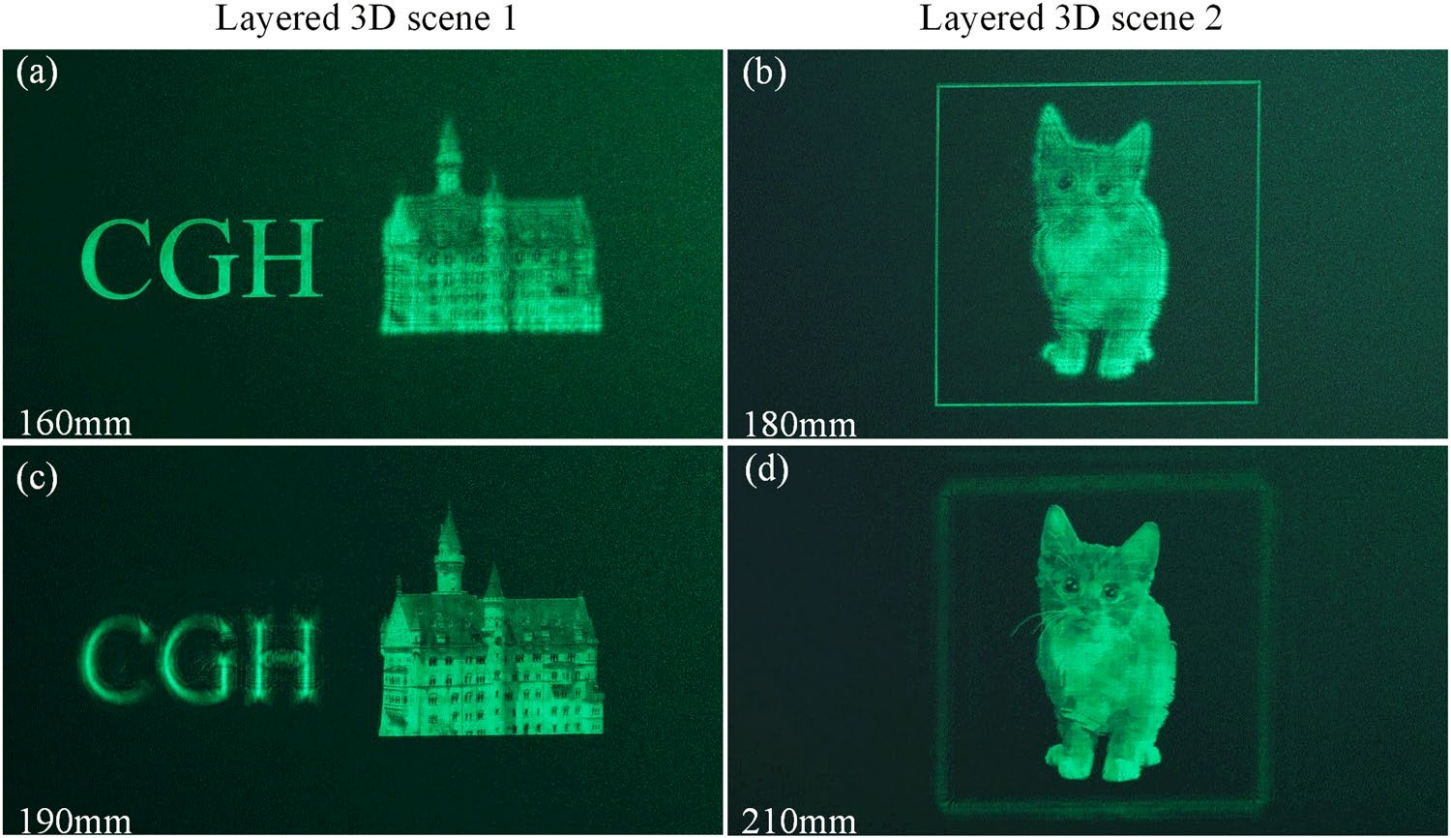
Optical reconstructions of layered 3D scenes.

In addition to generating CGHs for 2D reconstructions, we demonstrate the ability of our method to reconstruct layered 3D scenes in Fig. 3.

To generate 3D layered scene, each layer is combined with the predicted phase at the corresponding depth. After ASM to the CGH plane, the CNN is used to encode the synthetized complex amplitude. The detailed process is introduced in Methods section. The images are from DIV2K dataset and pixabay. The first 3D scene contains two objects placed at 160 mm and 190 mm, respectively. In Fig. 3a, the letter is in focus, while in Fig. 3c, the castle is in focus. The second 3D scene contains two objects placed at 180 mm and 210 mm, respectively. In Fig. 3b, the rectangle is in focus, while in Fig. 3d, the cat is in focus, demonstrating that our model can generate layered 3D scenes.

**Figure 4:**
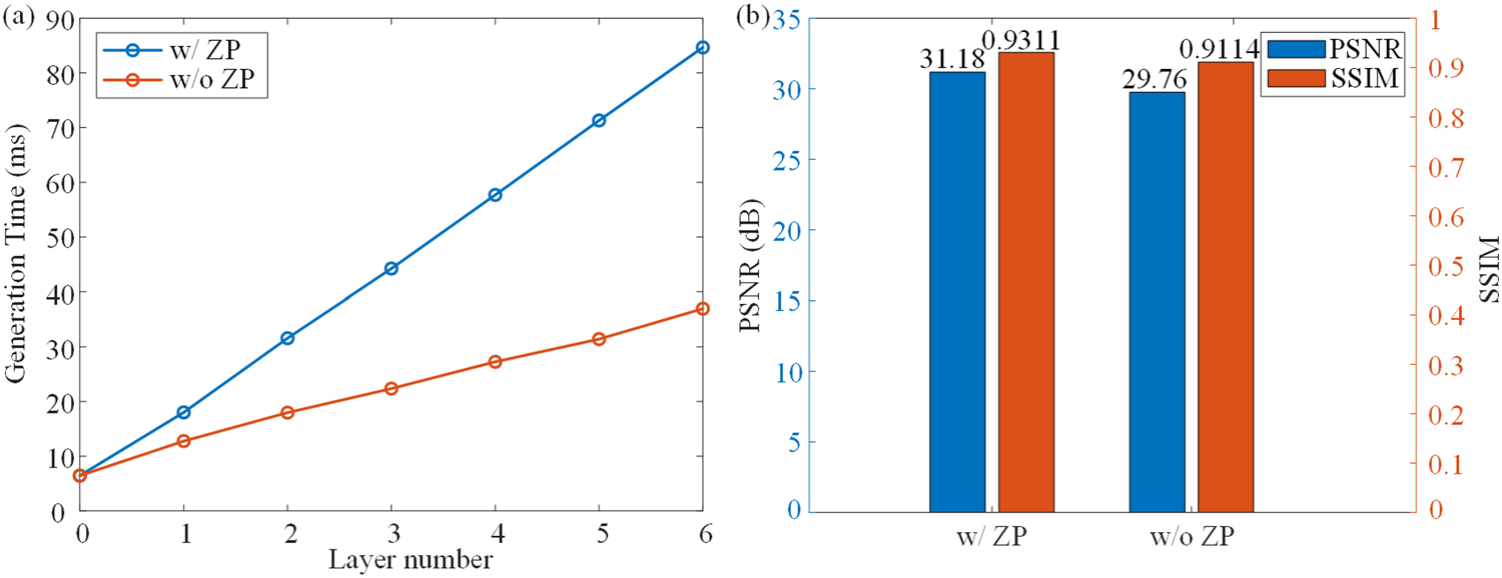
(**a**) Generation speed with the increase of layer number. (**b**) Average PSNR and SSIM.

**Figure 5:**
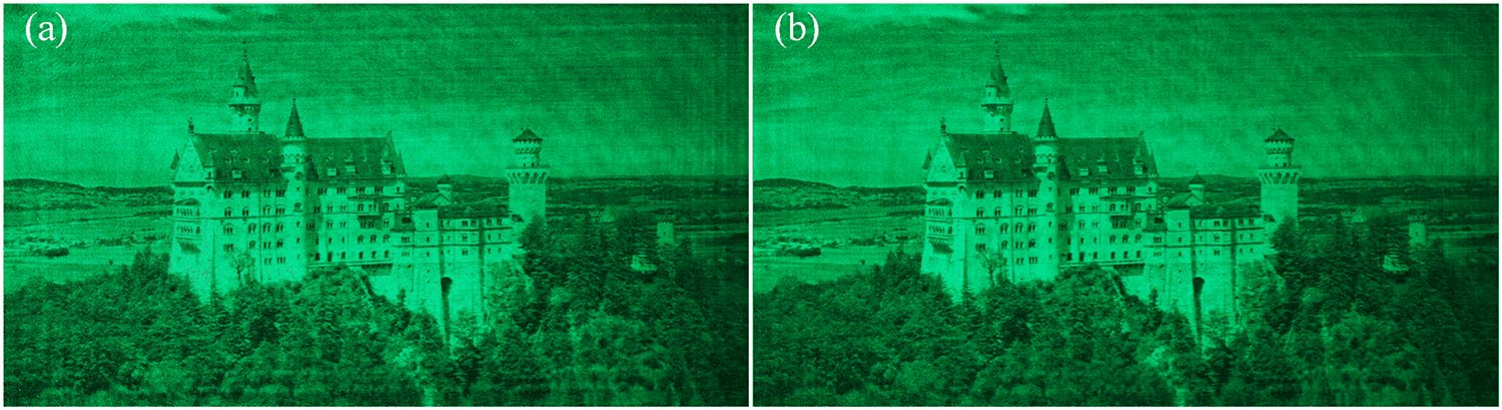
Optical reconstruction of (**a**) w/o ZP, (**b**) w/ZP.

### Generation speed and quality of proposed method

Figure 4 shows the generation speed for 4K CGH with varying layer numbers, as well as the average PSNR and SSIM of the 6 depth layers. The PSNR and SSIM are tested using 100 samples of the DIV2K validation dataset. 0 layer represents only running the encoding CNN. The total generation time comprises two components: diffraction calculation and CNN encoding. It shows that the encoding CNN takes up 6.47 ms to encode a 4K CGH using the complex amplitude on the CGH plane, and it takes around 12 ms to calculate the diffraction from one layer to the CGH plane. In the case of a single layer, the total generation time is 18.03 ms when utilizing an RTX3090 GPU. When switching to an Intel I7-12,700 CPU, the total generation time increases to 1926.95 ms. Zero-padding (ZP) ASM is used for more accurate diffraction calculation, but it slows down the ASM calculation. In Fig 4a, the red line represents that ZP is not implemented in the forward ASM, and it takes around 6 ms for each layer. Figure 4b shows the average PSNR and SSIM with ZP (w/ZP) and without ZP (w/o ZP) added in the forward ASM from the image plane to the CGH plane. Although the speed is improved without ZP, the quality is reduced. Figure 5 shows the optical reconstruction. The ringing artifact is more obvious if ZP is not used. Note that all our algorithms are implemented in python, using C++ may further accelerate diffraction calculation.

## Discussion

Here, we show detailed experiments on the learned phase. Using CNN for CGH encoding has been widely used in previous researches^11,19,22^. Our encoding CNN plays a similar role to encode the complex amplitude into CGH. The encoding CNN used is similar to our previous CCNN model^21^. The main difference is that we proposed a non-image-dependent initial phase for each depth layer. Thus, we firstly show a comparison of using and not using the proposed phase.

**Figure 6:**
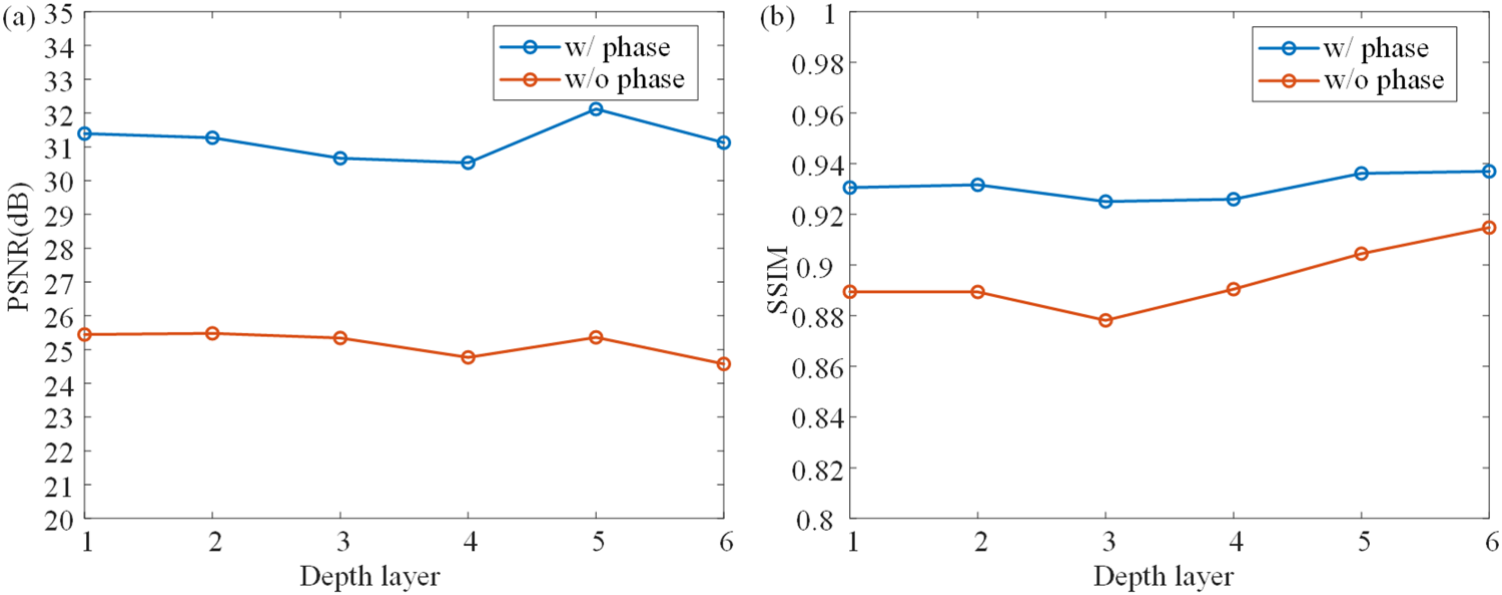
Average PSNR and SSIM of each depth layers using proposed method with learned phase (w/phase) and traditional method with zero phase (w/o phase).

Two models are trained to learn CGH generation, and the structure of the encoding CNN is the same. In contrast to the previous method that only uses CNN for encoding and sets zero initial phase (w/o phase)^19^, our method (w/phase) sets the initial phase as a learnable parameter. Both models are trained to generate 6 layers from 160 to 210 mm. 100 samples of DIV2K validation dataset are used to validate the models at different depth layer. As shown in Fig. 6, the PSNR of each layer is approximately 5 dB higher than that of previous method, and the SSIM is around 0.02 higher. Results show that the quality can be improved with our method. If the zero phase is used, the input amplitude still should be combined with the phase to create a complex-valued input for ASM, and the required time is the same, without considering the time to read the phase file from disk.

**Figure 7:**
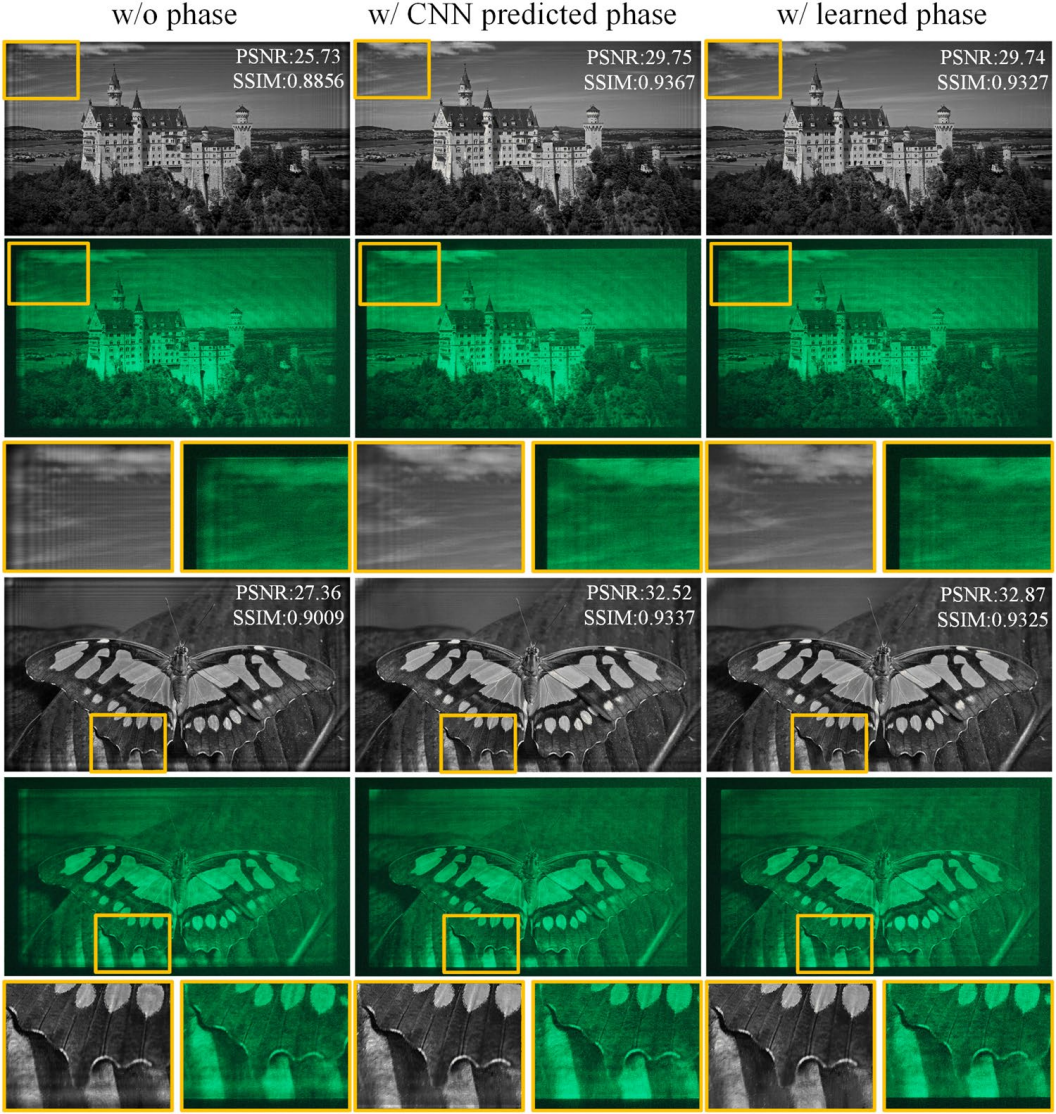
Numerical reconstructions and optical reconstructions of different methods.

It should be noted that HoloNet and our previous CCNN-CGH model used a CNN to predict an image-dependent initial phase. However, it is inefficient for a layered 3D scene if each layer is input into the CNN separately. To accelerate the generation, a non-image-dependent initial phase is presented to avoid using CNN to process the input image. To further compare the proposed method, zero phase, CNN predicted phase, we show the numerical and optical experiment in Fig. 7. The encoding CNN is the same, and the CNN used to predict initial phase is the same from our CCNN-CGH.

**Figure 8:**
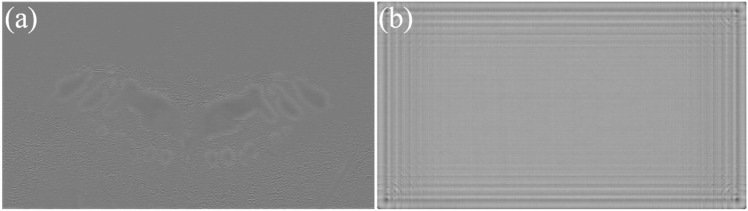
Initial phase for a target image using (**a**) CNN (**b**) proposed method.

In Fig. 7, we show the numerical and optical reconstructions obtained using different methods. The images are from DIV2K dataset. The generation speed for a 2D reconstruction is 18.03 ms, 54.82 ms and 18.03 ms, respectively. The proposed phase (w/learned phase) and zero phase (w/o phase) are not image-dependent, and they are applied to any target image. The CNN predicted phase is image-dependent, and it is generated according to the input image through a CNN. In the reconstruction, the CNN predicted phase produces better results than the zero-phase method. But the generation time is much longer because a CNN is used to process the target image. Although real-time reconstruction can be achieved in the case of 2D reconstruction, where only a 2D image is processed by a CNN. It would be inefficient in the case of layered scene where multiple images are input into the CNN separately. In contrast, our proposed method achieves a comparable quality while maintaining a fast generation time.

The initial phase for a target image using CNN and proposed method is shown in Fig. 8. For 2D reconstruction, we have introduced a learnable initial phase and trained it using natural images through gradient descent to enhance its adaptability to a wide range of images. This approach is referred to as ’non-image-dependent’ since prior research utilizes CNNs to predict the phase based on the input image^11^, resulting in a phase prediction that varies depending on the features of each input image. In contrast, our proposed initial phase is designed to be adaptable to any input image and remains independent of the specific features of the target image. This ’non-image-dependent’ initial phase accelerates the process of CGH generation by eliminating the need for phase prediction calculations based on the input image. For multiple layers, a distinct phase is assigned to each layer, and it can be regarded as training each phase independently at different depths.

In numerical and optical reconstructions, the ringing artifacts are more pronounced in the zero phase and CNN predicted phase methods, particularly at the edge of the reconstruction. The current CNN we use lacks position information input of the image pixels, and the CNN kernel is translation equivariant. The result of the proposed phase on the contrary vary according to the position. Incorporating positional information, in addition to image color information, may be a useful insight for future methods.

In summary, prior research has demonstrated the superiority of employing two separate CNNs for initial phase prediction and CGH encoding, as opposed to relying on a single CNN to predict the CGH directly^11^. Our method, however, challenges this conventional approach by showing that utilizing a learned phase can yield comparable quality results to CNN-predicted phases while significantly improving processing speed. Moreover, the inclusion of an additional phase in our approach outperforms previous methods that relied on a zero phase and a single CNN for encoding the complex amplitude of the CGH plane^19^.

**Figure 9:**
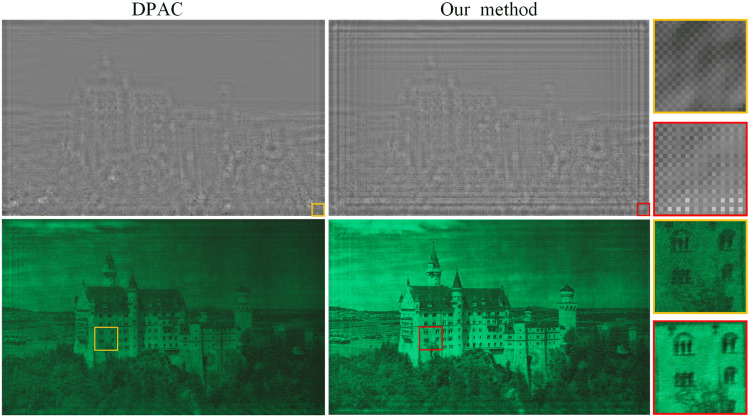
CGH and experimental result of DPAC and our method.

The encoding CNN converts the complex amplitude into a phase-only CGH, In Fig. 9, we compare between CNN encoding and Double-Phase Amplitude Coding (DPAC) . Existing research has demonstrated CNN’s superior performance over DPAC, particularly in terms of light efficiency^11^ and spatial shifting noises^19^. Our experimental result is shown in Fig. 9. Both optical reconstructions were captured under identical camera settings, highlighting the advantage of CNN in terms of light efficiency. The encoded CGHs are also presented for visual comparison. CNN-encoded CGH exhibits a checkerboard-like pattern, but the rule is different compared to DPAC. In our simulation, The PSNR is 29.73 dB using our method, and 14.9 dB using DPAC. The SSIM is 0.93 and 0.85 for our method and DPAC, respectively.

At last, our method has limitations that need to be addressed in further research.

### Natural defocus blur

In our method, only the focused part is optimized, and it can be observed that artifacts occur when the images are defocused. Those artifacts will affect the quality of 3D scenes. Recent progress for defocus optimization such as time-multiplex and diffraction-engineered holography can be combined to further optimize the quality of 3D scenes^17,26^.

### Occlusion culling for 3D scenes

Our method has not been optimized to reconstruct 2D+D scenes with continue depth currently. In the depth-map CGH, artifacts will be observed in the discontinuous edges of each depth layer, which can negatively impact the quality of the 3D displays. As the depth gap of adjacent layers increases, the presence of inter-layer edge artifacts becomes more pronounced. In our approach, each layer has a depth gap of 10 mm, resulting in a total depth range of 50 mm. Displaying 3D scenes with continuous depth will face severe artifacts. Thus, only 3D scenes composed of different objects are presented. Recent methods for smoothing inter-layer edge artifacts can be used to optimize the 3D reconstruction^27^.

### Accurate wave propagation

Our method is optimized with the ideal ASM, which may not accurately simulate the wave propagation in actual displays. Recent advances in learned diffraction models, such as Neural 3D Holography and Time-multiplexed Neural Holography, can be used for accurate wave propagation and as a loss function^16,17^.

### Color display

Our method only calculates a single color CGH at a time. Color display can be achieved by training three models for three color channels separately. But the generation time is three times. Future work can be done for generate color CGH using a model with multiple output channel such as Ref.^14^.

## Methods

### Architecture of the proposed method

Figure 1 shows the training process of our model. At first the input amplitude *A* is assigned a random depth layer *d* and the corresponding phase $\varphi _{d}$. The complex amplitude ${C}_{d}$ is:

1 $$\begin{aligned} C_d=A \exp \left( i \varphi _d\right) \end{aligned}$$

Then ASM is used to calculate the diffraction from the image plane to the SLM plane. The complex amplitude ${D}_{d}$ in the SLM plane is calculated by:

2 $$\begin{aligned}{} & {} D_d=\mathcal {F}^{-1}\left[ \mathcal {F}\left( C_d\right) \cdot H_d\right] \end{aligned}$$

3 $$\begin{aligned} H_d=\left\{ \begin{array}{ll }\exp \left( i k z_d \sqrt{1-\lambda ^2 f_x^2-\lambda ^2 f_y^2}\right), & \quad \text{ if } \sqrt{f_x^2+f_y^2}<\frac{1}{\lambda } \\ 0, & \quad \text{ otherwise } \end{array}\right. \end{aligned}$$

where $\mathcal {F}$ represents the two-dimensional fast Fourier transform, and $\mathcal {F}^{-1}$ represents the reverse two-dimensional fast Fourier transform. *k* is the wave number, and ${z}_{d}$ is the distance of propagation. $\lambda$ is the wavelength, ${f_x}$ and ${f_y}$ are the spatial frequencies in the x and y directions respectively. We used zero-padding and band-limited ASM for more accurate diffraction calculation^28,29^.

If the CGH is generated for a 2D reconstruction, ${D}_{d}$ is directly input into the encoding CNN. Note that using a CNN to generate 2D scenes for different depth layers has been proposed by Ryu et al.^30^, but their method is not optimized for layered CGH generation because the CNN need to be used multiple times for each layer. In our method, the CNN is used once for multiples layers. For layered 3D scenes, the complex amplitude in the CGH plane from different each layer is added before encoding. For example, if the 3D scene is composed of 2 layers ${d_1}$ and ${d_2}$, the complex amplitude in the CGH plane is calculated by adding the complex amplitude ${D}_{d1}$ and ${D}_{d2}$ propagated from each layer:

4 $$\begin{aligned} D_{total}=D_{d1}+D_{d2} \end{aligned}$$

Then the CNN is used to encode the complex amplitude to CGH. The output of the CNN is also a complex amplitude. Its phase p is used for phase-only CGH. The simulated complex amplitude ${D}^{\prime }$ modulated by the phase-only SLM is:

5 $$\begin{aligned} D^{\prime }=\exp \left( i p\right) \end{aligned}$$

Then ASM is used to simulate the reconstruction, the complex amplitude ${C}^{\prime }$ of the reconstruction is:

6 $$\begin{aligned} C^{\prime }=\mathcal {F}^{-1}\left[ \mathcal {F}\left( D^{\prime }\right) \cdot \bar{H}_d\right] \end{aligned}$$

$\bar{H}_{d}$ is the conjugate of the original transfer function ${H}_{d}$ , because the direction is opposite. After the simulation of reconstruction, MSE loss is used as the loss function between original amplitude A and the reconstructed amplitude. Perceptual loss function and phase constraint can also be used to further enhance the quality^31,32^. Adam optimizer is used to optimize the CNN and initial phase.

**Figure 10:**
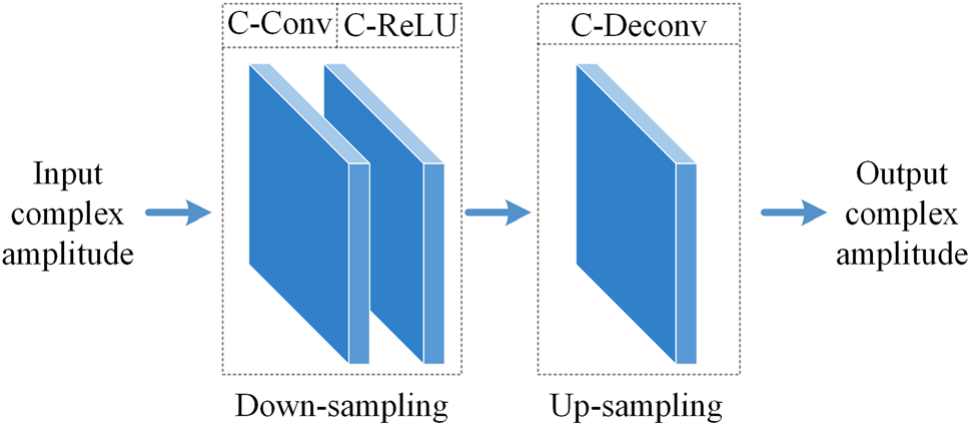
Detailed structure of the encoding CNN.

The architecture of the encoding CNN is shown in Fig. 10. Here, we use the same Down-sampling and Up-sampling block as our previous CCNN-CGH network^21^. The encoding CNN uses only one complex-valued convolution (C-Conv) layer, ReLU (C-ReLU) layer and deconvolution (C-Deconv) layer. The input is the complex amplitude after ASM from the image plane. The convolution kernel size of C-Conv is 3 × 3, and the stride is 2. The channel number is 18 after the C-Conv. The convolution kernel size of C-Deconv is 4 × 4, and the stride is 2. The output is a complex amplitude and its phase is used as the phase-only CGH.

The parameter number in a real valued CNN is calculated by:

7 $$\begin{aligned} Params=\left( S \cdot C_{\text{ in } }+1\right) \cdot C_{\text{ out } } \end{aligned}$$

where *S* is the size of the convolution kernel, ${C}_{in}$ is the input channel number, 1 is the bias of the kernel. ${C}_{out}$ is the output channel number. In C-Conv, the parameters are doubled. Thus, the parameter number for our encoding CNN is 938. Compared with millions of parameters in previous methods. The number of parameters is very small in our CNN.

### Experiment setup

DIV2K training dataset is used to train the neural networks. The training is performed on a computer with an Intel i7-12700K processor, 32GB of RAM, and an NVIDIA RTX 3090 GPU with 24GB memory, running on the Windows 10 operating system. The learning rate is 0.001. It takes approximately 1.5 hour to train our model for 50 epochs. The GPU memory occupation is 5.9 GB for 1 layer and 8.8GB for 6 layers.

**Figure 11:**
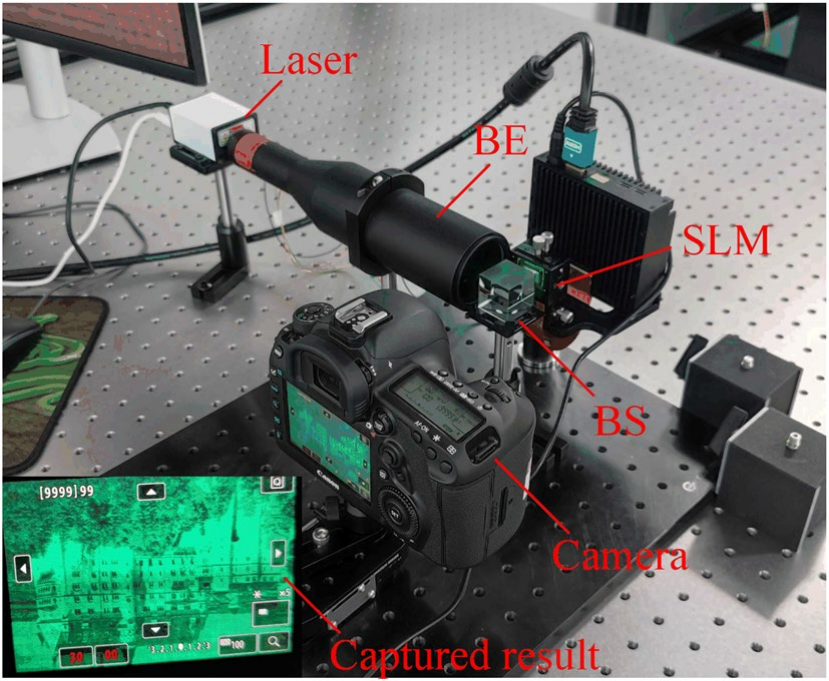
Optical setup.

The optical setup of the holographic display prototype is shown in Fig. 11. Coherent light is provided by a laser and the wavelength is 520 nm. A beam expander (BE) is used to expand the laser. The optical path is adjusted with a beam splitter (BS). An SLM (HDSLM36R) is used with a pixel pitch of 3.6 μm and a resolution of 3840*2160 to reflect and modulate the incident light. The holographic reconstruction is captured with a Canon EOS 5D Mark IV camera.

## Conclusion

In summary, a novel approach for generating 3D CGHs is presented. A non-image-dependent learned phase is proposed for each target depth layers. After diffraction from the image planes to the CGH plane, a CNN is used to encode the CGH by the complex amplitude on the CGH plane. We show that our method can generate high-quality 4K CGH in real-time, and the CGH can be generated for different depth layers and layered 3D scenes. The generation time is 18 ms for 2D reconstruction, and increased by 12 ms for each additional layer for 3D scenes. The average PSNR is above 30dB in our target depth layers. Our method can be applied to real-time 4K holographic displays.

## Data Availability

The code is available in our GitHub repository, https://github.com/flyingwolfz/RT-4K-CGH.
